# Effects of conditioned media derived from human Wharton’s jelly mesenchymal stem cells on diabetic nephropathy and hepatopathy via modulating TGF-β and apelin signaling pathways in male rats

**DOI:** 10.1186/s12902-023-01535-8

**Published:** 2024-01-05

**Authors:** Zeinab Karimi, Gholamreza Daryabor, Fatemeh Masjedi

**Affiliations:** 1 Shiraz Nephro-Urology Research Center, Shiraz University of Medical Sciences, Shiraz, Iran; 2 Autoimmune Diseases Research Center, School of Medicine, Shiraz University of Medical Sciences, Shiraz, Iran

**Keywords:** Diabetes mellitus, Wharton’s jelly mesenchymal stem cells, Conditioned media, Apelin, Transforming growth factor-beta

## Abstract

**Background:**

Diabetic nephropathy and hepatopathy are health problems described by specific renal and hepatic structure and function disturbances. The protective effects of the stem cell secretome have been shown in several kidney and liver diseases. The current study aims to evaluate the capability of conditioned media derived from human Wharton’s jelly mesenchymal stem cells (hWJ-MSCs-CM) to alleviate diabetic complications.

**Methods:**

Twenty Sprague Dawley rats were made diabetic through injection of STZ (60 mg/kg, i.p.). At week 8, diabetic rats were divided into two groups: treated [DM + hWJ-MSCs-CM (500 µl/rat for three weeks, i.p.)] and not treated (DM). At the 11th week, three groups (control, DM, and DM + hWJ-MSCs-CM) were kept in metabolic cages, and urine was collected for 24 h. The serum samples were maintained for measuring fasting blood glucose (FBG) and kidney and liver functional analysis. The left kidney and liver parts were kept at -80 °C to assess apelin and transforming growth factor-beta (TGF-β) expression. The right kidney, pancreas, and liver parts were used for histopathologic evaluation.

**Results:**

DM was detected by higher FBG, microalbuminuria, increased albumin/creatinine ratio, and pancreas, renal, and hepatic structural disturbances. Diabetic hepatopathy was determined by increasing liver enzymes and decreasing total bilirubin. The TGF-β gene expression was significantly upregulated in the diabetic kidney and liver tissues. Apelin gene expression was significantly downregulated in the diabetic liver tissue but did not change in kidney tissue. Administration of hWJ-MSCs-CM improved renal and hepatic functional and structural disturbances. Moreover, CM therapy significantly decreased TGF-β expression and enhanced apelin expression in the kidney and liver tissues.

**Conclusion:**

Human WJ-MSCs-CM may have protective effects on diabetic renal and hepatic complications. These effects may happen through the regulation of TGF-β and apelin signaling pathways.

## Background

Individuals with type 1 and type 2 diabetes mellitus (DM) are influenced by different genetic factors, but both are prone to developing complications such as retinopathy, peripheral neuropathy, nephropathy, and hypertension [1, 2].

According to most estimates, diabetes is the leading cause of liver disease in the United States. It has been reported that liver disease is a leading cause of death in type 2 diabetes [3]. Consequently, patients with diabetes are more likely to be diagnosed with liver disease, and conversely, patients with liver disease are at greater risk of developing diabetes. Patients with type 2 diabetes are likely to suffer from a broad spectrum of liver disease, including abnormal liver enzymes, non-alcoholic fatty liver disease (NAFLD), cirrhosis, and acute liver failure [4]. Chronic mild elevations of transaminases are common in patients with type 2 diabetes. On the other hand, liver failure is not treated with an equivalent method, such as hemodialysis or retinal photocoagulation [5]. As such, although diabetic hepatopathy is potentially less common than glomerulopathy, retinopathy, and neuropathy, it could be considered as one of the target organ conditions associated with diabetes.

There is a pathogenic mechanism related to hyperglycemia-induced glucose influx that sequentially activates glycation end products (AGEs), thereby altering the structure of the extracellular matrix (ECM) and making it resilient to degradation [6]. Finally, AGEs promote the production of extracellular transforming growth factor (TGF), leading to ECM accumulation and fibrosis development [7, 8]. Emerging evidence suggests that the inflammatory pathway plays a major role in developing diabetic nephropathy (DN) [9]. In patients with type 1 and type 2 diabetes, the plasma level of TGF-β1 is significantly increased, and this value is even higher in people with DN [10, 11]. Moreover, there is a strong correlation between the plasma and urinary levels of TGF-β1 and the severity of renal dysfunction in patients with DN [12, 13], suggesting the involvement of renal TGF-β in the pathogenesis of DN.

Apelin, an adipokine peptide discovered recently, is protective against diabetic complications. According to previous studies, diabetics may also demonstrate apelin resistance similar to insulin or leptin resistance [14]. It has been reported that plasma apelin levels are reduced in newly diagnosed type 2 diabetic patients, consistent with the observation that plasma apelin levels rise after rosiglitazone and metformin therapy for 14 weeks, and glycemic profiles improve [15]. Moreover, another research indicated that apelin injection reduces blood glucose levels, plasma insulin, and blood pressure in type 2 diabetic rats with hypertension [16, 17]. In human and rodent islets, apelin is expressed in β and α cells, as well as in a sub-population of PP (pancreatic polypeptide-producing) cells. The apelin receptor (APJ) is expressed in human islets and a rat insulinoma cell line [18], suggesting that apelin plays a paracrine or autocrine role in the function of pancreatic islets [19].

Based on other findings, apelin alleviates diabetes-induced albuminuria in type 1 diabetes animal models by reducing renal inflammation and hypertrophy [20, 21]. There is also evidence that apelin-13 alleviates DN by increasing the production of nitric oxide (NO) and improving renal fibrosis [22]. Furthermore, apelin-13 suppresses the injury-induced elevation of TGF-β1 and apoptosis associated with renal ischemia/reperfusion (I/R) injuries [23]. It has also been shown that the apelin peptide is expressed in healthy human livers and implicated in preventing hepatic fibrosis and cirrhosis [24].

Interestingly, an increasing body of research indicates that stem cells exert their therapeutic effects primarily through paracrine signaling and secreted extracellular vesicles and exosomes [25, 26]. This belief is supported by several studies reporting beneficial therapeutic effects of conditioned media derived from human Wharton’s jelly mesenchymal stem cells (hWJ-MSCs-CM) [27–29]. Several recent studies have found that conditioned media therapy effectively reduces inflammation, apoptosis, and fibrosis in acute and chronic kidney and liver disease [30–33].

Considering the involvement of apelin and TGF-β signaling pathways in diabetic nephropathy and liver damage, as well as the ameliorating effects of MSCs-conditioned media therapy on liver and kidney diseases, the present study aims to investigate the protective effects of hWJ-MSCs-CM through the mentioned signaling pathways on the renal and hepatic complications of diabetes.

## Materials and methods

### Primary culture of human Wharton’s jelly mesenchymal stem cells (hWJ-MSCs)

Umbilical cord samples were collected from the cesarean delivery of full-term infants. The Ethics Committee of Shiraz University of Medical Sciences approved this study (IR.SUMS.REC.1400.099), and written informed consent was obtained from the parents.

The tissue samples were transferred to the lab in cold phosphate-buffered saline (PBS) (Sigma-Aldrich, MO, St. Louis, USA) containing 100 U/mL penicillin (Sigma-Aldrich) and 100 µg/mL streptomycin (Sigma-Aldrich) and washed three times. Then, the arteries were removed, the umbilical vein was opened, and the endothelium was crushed using a sterile blade [34]. Then, the umbilical cords were cut into small explants about 5 mm each and placed in the dishes. After 15 min, α-MEM (Gibco BRL, life technology, Germany) containing 10% fetal bovine serum (FBS) (Gibco BRL), 1% L-glutamine (Sigma Aldrich), and 100 U/mL penicillin (Sigma-Aldrich), 100 µg/mL streptomycin (Sigma-Aldrich) were added to the culture plates [35].

### Characterization of hWJ-MSCs

The cell suspension was adjusted at a concentration of 1 × 10^6^ cells/mL in 10% FBS/PBS as the blocking solution for 20 min. Then, the cells were labeled with FITC-conjugated anti-CD90 and CD144, phycoerythrin-conjugated anti-CD73, and CD34 antibodies (all from Abcam, UK) for 30 min [36]. The frequencies of positive cells were evaluated by a FACS-calibrated instrument (BD Biosciences, San Diego, CA, USA) and analyzed using FlowJo™ Software (Version 10.5.3, BD Biosciences).

### Adipogenic and osteogenic differentiation of hWJ-MSCs

Human WJ-MSCs were induced to differentiate into osteocytes and adipocytes by exposure to osteogenic (MACS, Germany) and adipogenic media (Stem Cell Technologies Inc., Canada) for 4 and 3 weeks, respectively. The culture media were replaced twice a week. Then, the hWJ-MSCs differentiated toward osteogenic lineage were fixed with 4% paraformaldehyde and stained with Alizarin Red S (Sigma, USA). The cells were stained by Oil Red O (Sigma, USA) to demonstrate adipogenic differentiation [36].

### Preparation of hWJ-MSCs-conditioned media

Based on the Sanie-Jahromi et al. [27] method, 1 × 10^6^ hWJ-MSCs at the third passage were seeded in a T75 tissue culture flask. The confluent cells were fed with serum-free medium and cultured for 72 h. The medium was collected and centrifuged at 3000 g for 4 min at room temperature. The supernatant was filtered by a 0.22-mm filter, used as conditioned media (CM) of hWJ-MSCs, and stored at -80 °C.

### Experimental animals and protocol

All the procedures for studies on animals were approved by the Ethics Committee of Shiraz University of Medical Sciences (IR.SUMS.REC.1400.099). All applicable international, national, and/or institutional guidelines for the care and use of animals were followed. All methods are reported in accordance with ARRIVE guidelines.

Male Sprague-Dawley rats (weight 200–250 g) were purchased from the Shiraz University of Medical Sciences animal center. They were housed in individual cages at 22–25 °C for a 12 h/12 h light/dark cycle. Food and water were available for animals unless otherwise stated. Ten rats in each group were used in line with the sample size calculation in a previous study [35].

After one week of adaptation, the rats were randomly divided into three groups (n = 10 for each group): the control group and twenty diabetic rats. We used a well-established rat model of DM induced by STZ, as described [37]. Briefly, induction of diabetes was performed by a single intraperitoneal injection of STZ (Sigma-Aldrich) (60 mg/kg was dissolved in the fresh 0.1 M citrate buffer). The control group received an equal volume of vehicle treatment. After blood sampling from the tail vein, fasting blood glucose (FBG) was measured using a commercially available kit (Pars Azmun, Iran). Diabetes status was confirmed by a random glucose level higher than 16.7 mmol/L for three consecutive days [38].

Then, the rats with diabetes mellitus (DM) were randomly divided into two groups: the hWJ-MSCs-CM treated group (DM + hWJ-MSCs-CM) and the not-treated group that received pure α-MEM as a vehicle (DM group). Human WJ-MSCs-CM was given (i. p.) 500 µL per rat daily for three weeks [39].

Three weeks after hWJ-MSCs-CM treatment (at the end of 11th weeks, on the last day of the experimental period), all rats were kept in metabolic cages (Tecniplast, Italy), and their urine was collected over 24 h to measure volume and protein concentration. Diabetic nephropathy was detected by 24 h urinary albumin excretion (UAE > 30 mg/day) and urine albumin/creatinine ratio (UAC > 300 mg/g) [40]. Food and water intake and body weight were also recorded. Urine samples were centrifuged to eliminate any suspended elements, and the supernatant was used to measure 24 h urine albumin and creatinine levels. Then, the rats were weighed and anesthetized with ketamine (Alfasan Int., Woerden, Netherlands) and xylazine (Alfasan Int.) (50 and 10 mg /rat’s weight, respectively). A blood sample was taken from the tail vein of the animals. After centrifugation, the serum was separated and stored at -20 °C to assess functional organ indices. Subsequently, the rats were sacrificed, and their pancreas, liver, and kidneys were immediately removed. Their kidneys decapsulated and longitudinally divided into two sections. The left kidney and one liver lobe were kept at -80 °C for molecular assay. The right kidney, one lobe of the liver, and pancreas were maintained in 10% formalin (Merck & Co., Inc., Rahway, NJ, USA) for Hematoxylin-Eosin (H & E) (Sigma-Aldrich) staining.

### Measurement of functional indices of kidney and liver

The values of FBG were assessed using a commercially available kit (Pars Azmun, Iran). Serum and urinary creatinine, as well as aspartate aminotransferase (AST), alanine aminotransferase (ALT), alkaline phosphatase (ALP), lactate dehydrogenase (LDH), and total bilirubin, were measured using an autoanalyzer (Prestige, Biolis 24I, Japan) in the laboratory of Namazi Hospital, Shiraz, Iran. Serum and urinary creatinine and urine flow rate (V^0^) were used to determine the glomerular filtration rate (GFR = urine creatinine * urine flow rate/serum creatinine). In addition, urine albumin concentration was assayed with a kit (Pars Azmun, Iran) to calculate the urine albumin exertion (UAE) and urine albumin/creatinine ratio (ACR) [41].

[UAE (mg/24 h)= [urine microalbumin (mg/mL) * urine output (mL/24 h)]/1000]

[ACR = UAE (mg/24 h)/urine creatinine (g/L)]

### Gene expression analysis

Tissue samples (40 mg) were homogenized using TissueLyser LT (QIAGEN), and total RNA was extracted using an RNA extraction kit (Yekta Tajhiz, Iran) according to the manufacturer’s protocol. The concentration and purity of RNA samples were assessed using a UV spectrophotometer (Pico 100 µl Spectrophotometer, Picodrop Limited, Hinxton, United Kingdom) by measuring the absorbance at 260 and 280 nm. RNA was then reverse-transcribed using an AddScript cDNA Synthesis Kit (Addbio, Korea) according to the manufacturer’s protocol. Real-time PCR was performed in triplicate using the StepOne Real-Time PCR System (Applied Biosystems, ThermoFisher Scientific, Foster City, CA, USA). Details of the selected genes, primer sequences, and PCR conditions are listed in Table 1.

**Table 1:** Studied genes, primer sequences, and PCR conditions

Transcripts	Primer	Primer Sequences (5’-3’)	Thermocycling Condition	PCR Mix
***GAPDH***	Forward	AGTGCCAGCCTC GTCTCATA	95 °C/10 min, 40 cycles at 95 °C/15s, 58 °C/20s, and 72 °C/30 s	SYBR green Master Mix (10 µl; 2x concentration), Forward primers: 0.8 µl and 10 pmole, Reverse primers: 0.8 µl and 10 pmole, Nuclease-free DEPS Water: 6.4 µl, Template cDNA: 2 µl
	Reverse	GAGAAGGCAGCCCTGGTAAC		
***TGF-β***	Forward	TGACATGAACCGACCCTTCC	95 °C/10 min, 40 cycles at 95 °C/15s, 60 °C/20s, and 72 °C/30 s	
	Reverse	TGCCGTACACAGCAGTTCTT		
***Apelin***	Forward	CTCTGGCTCTCCTTGACTGC	95 °C/10 min, 40 cycles at 95 °C/15 s, 59 °C/20s, and 72 °C/30s	
	Reverse	TCGAAGTTCTGGGCTTCACC		

Briefly, 2 µl cDNA, 10 pM of forward and reverse primers, and 10 µl Real Q plus 2X Master Mix Green (Ampliqon, Stenhuggervej, Denmark) were used in a total volume of 20 µl. The PCR parameters were as follows: 95 °C for 15 min followed by 45 cycles of denaturation (95 °C for 30 s), annealing (60 °C for 30 s), and extension (72 °C for 30 s). The relative expression level of target genes in each sample was normalized against GAPDH using the 2^−ΔΔCT^ method and was shown as relative fold change (RFC) compared to the control sample.

### Histopathological analysis of pancreas

To study the histopathological changes, pancreas samples were placed in 10% buffered formalin, and 5 μm thick sections were prepared for H&E staining.

Based on Masjedi et al. [42] method, pancreatic-stained sections were evaluated quantitatively (morphometry) and qualitatively (morphology). For quantitative analysis of the pancreas, the following variables were assessed:

I: The average number of pancreatic islets was calculated in 10 parts of the pancreatic parenchyma in each section (equivalent to 10 fields of 10x objective lens of the light microscope) and 20 fields in each group.

II: Mean diameter of the pancreatic islet was measured in 6 islets in each section and a total of 12 islets in each group using ImageJ software (NIH, Bethesda, Maryland, USA) and calculated with the following formula:

Mean diameter = √ l × b × magnification, where l was the length and b was the breadth of the islets.

### Histopathological analysis of kidney

The right kidney samples were embedded in 10% buffered formalin to study renal histopathological changes, and 5-µm thick sections were prepared for H&E staining. Ten randomly selected non-overlapping fields were assessed in each renal slide by light microscope, and the degree of glomerular and tubular damage was quantified, as previously described [43, 44].

### Histopathological analysis of liver

Using a 10% formalin solution for 24 h, a histological inspection of the liver tissue was performed, and the fixative was extracted by washing overnight with flowing tap water. Methyl benzoate was used to clean the tissues, and they were embedded in paraffin after dehydration by a graduated sequence of alcohols. Sections were cut at 5 μm thickness by a microtome and stained with H&E.

According to previous studies [45, 46], histopathological scoring research was conducted. The evaluation was represented as the sum of the individual grades of 1 (minimum), 2 (mild), 3 (moderate), and 4 (marked) for each of the following liver parameters: necrosis of hepatocytes, cholestasis, hyperplasia, and shift in hepatocyte fat.

### Statistical analysis

The normality of data distribution was checked using the Shapiro–Wilk test. Multiple comparisons between groups were done using one-way ANOVA, followed by Tukey’s post-hoc test. Results are presented as means ± SEM. A P-value < 0.05 was considered as significant level. Statistical analyses were performed using GraphPad Prism software (version 9.0, Inc. La Jolla, CA, USA).

## Results

### Characterization and differentiation of hWJ-MSCs

**Fig. 1:**
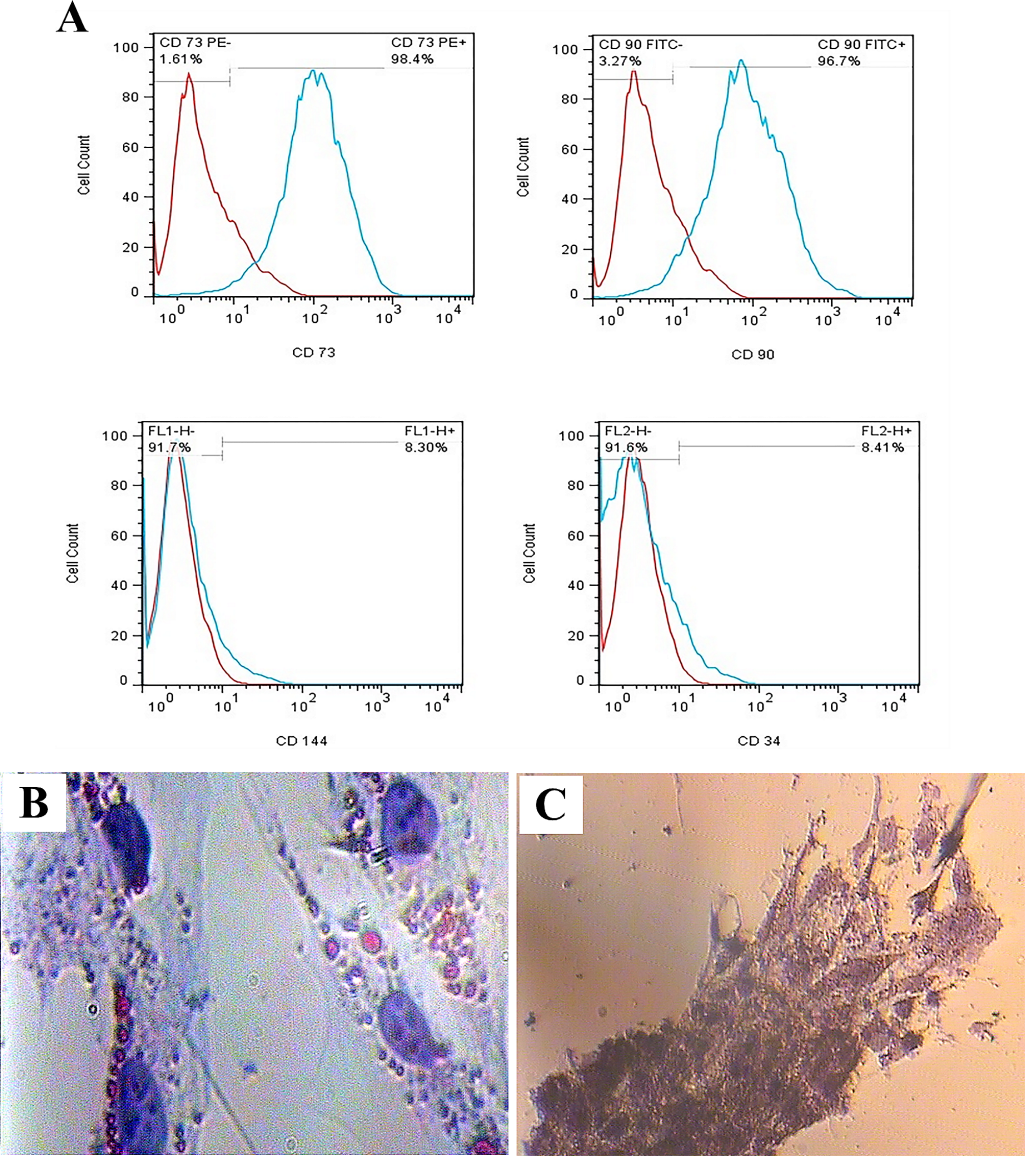
The flow cytometry revealed the frequency of hWJ-MSCs that reacted to CD90 and CD73 was high, whereas the frequency of the cells that responded to CD34 and CD144 was negligible (**A**), Oil red O staining showed that the cells stored lipid droplets in the presence of adipogenic medium (**B**), and Alizarin red S showed that the cells deposited Ca^2+^ in the presence of osteogenic medium (**C**)

The flow cytometry analysis indicated that hWJ-MSCs were positive for the MSC surface markers, such as CD90 (96.7%) and CD73 (98.4%), and negative for CD34 (8.41%) and CD144 (8.30%) markers (Fig. 1A). Furthermore, the Oil Red O and Alizarin Red S staining confirmed the capability of the cells to differentiate toward adipogenic and osteogenic cell lineages, respectively (Fig. 1B and C).

### Induction of in vivo model of DM using STZ

**Fig. 2:**
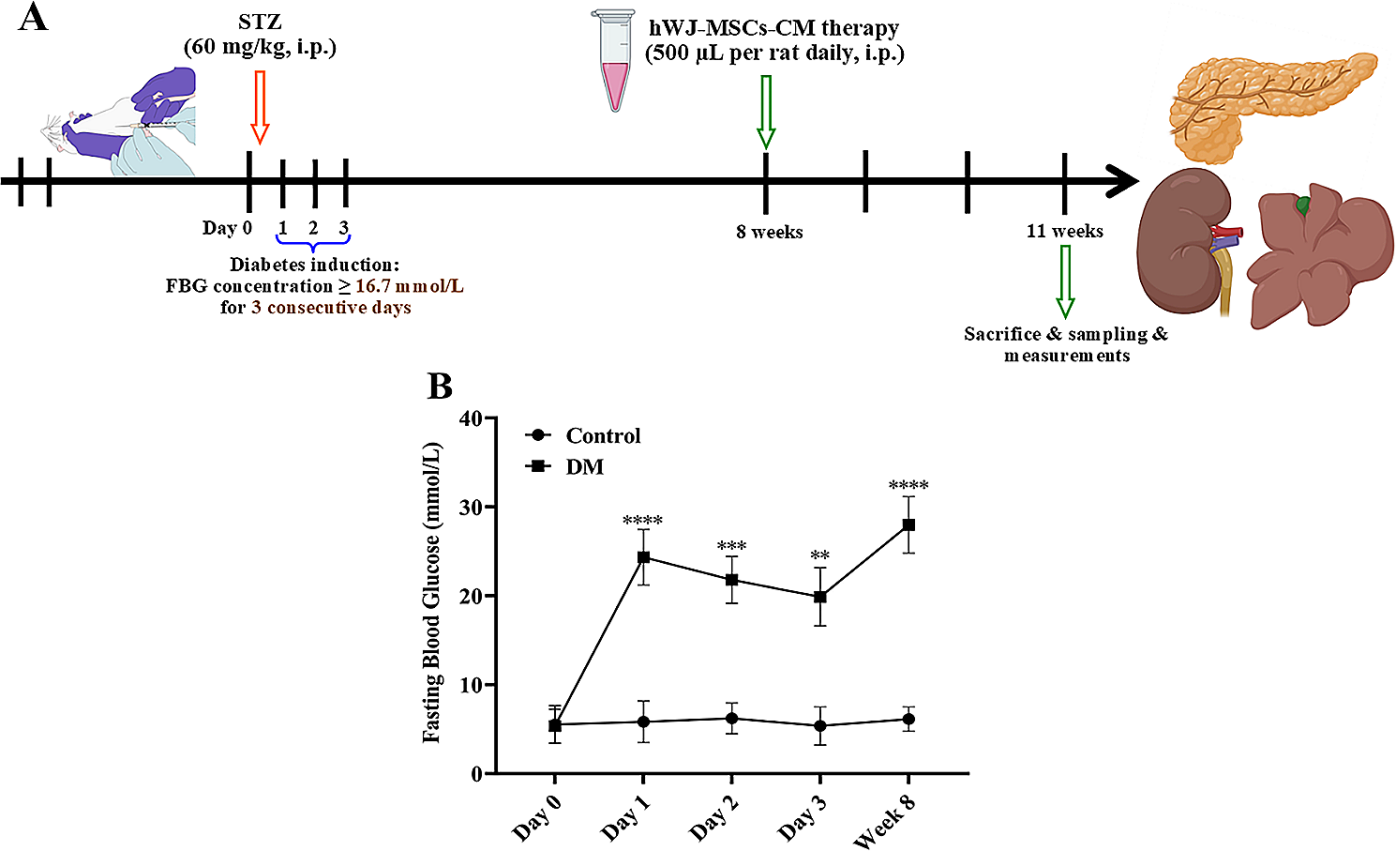
Timeline and protocol of conditioned media therapy as well as the identification of rat DM model. The timeline of rat treatment from day 0 to week 11 (**A**) and FBG concentration curve on three consecutive days after STZ injection (**B**). Data are expressed as mean ± SEM (n = 10). ****P < 0.0001, ***P < 0.001, and **P < 0.01 represent significant differences with controls. DM = Diabetic mellitus; hWJ-MSCs-CM = Conditioned media derived from human Wharton’s jelly mesenchymal stem cells; FBG = Fasting blood glucose; STZ = Streptozotocin

To explore the therapeutic effect of hWJ-MSCs-CM on DM, we established a rat model of DM induced by STZ injection. Animals were sacrificed after three weeks of treatment, and specimens were collected for further analysis (study protocol, Fig. 2A). The FBG of the DM group after STZ injection was greater than 16.7 mmol/L and was significantly (Day 1: P < 0001, Day 2: P = 0.0005, Day 3: P = 0.0037) higher than that of the control rats (Fig. 2B).

### Effects of hWJ-MSCs-CM on diabetic indices and survival variables

At the end of the experiment, significant increases (P < 0.0001) were observed in FBG, UAE, and ACR in the DM group. Compared with the control group, the rats in the DM group had polyuria and polydipsia. However, these animals had lower body weights. Administration of hWJ-MSCs-CM (for three weeks) significantly alleviated all indices of diabetes and the survival variables compared to the DM group. However, all variables in the DM + hWJ-MSCs-CM group still had significant differences from the control group (Table 2).

### Effects of hWJ-MSCs-CM on renal function variables

**Fig. 3:**
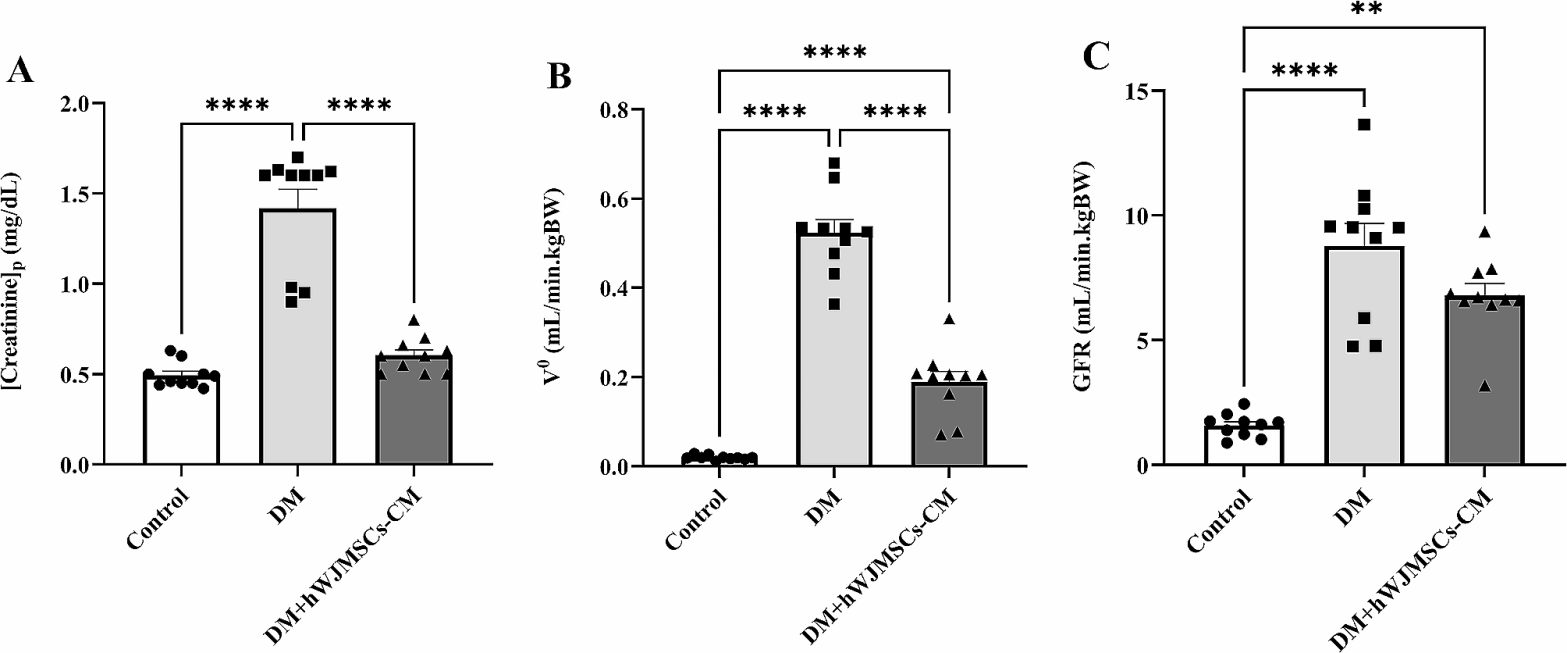
The effects of three weeks of hWJ-MSCs-CM treatment (500 µL per rat, i.p.) on serum creatinine (**A**), V^0^ (**B**), and GFR (**C**) in rats with DM. Data are expressed as mean ± SEM (n = 10). ^****^P < 0.0001 represents significant differences between experimental groups. DM = Diabetic mellitus; hWJ-MSCs-CM = Conditioned media derived from human Wharton’s jelly mesenchymal stem cells; V^0^ = urine flow rate; GFR = glomerular filtration rate

Serum creatinine levels significantly (P < 0.0001) increased in diabetic rats, which was related to the significant disturbance in renal functions (Fig. 3A). Moreover, a significant (P < 0.0001) increase in V^0^ was observed in the DM group (Fig. 3B). The GFR level was also significantly (P < 0.0001) higher in the STZ-induced diabetic rats than those that controls (Fig. 3C). Intra-peritoneal injection of hWJ-MSCs-CM decreased all parameters of renal dysfunction in the DM + hWJ-MSCs-CM group compared with the diabetic group (P < 0.0001). However, the GFR level in the treatment group still significantly differed from the control group (P = 0.007).

**Table 2:** State of diabetic indices and survival variables in the experimental groups

Experimental Groups Variables	Control	DM	DM + hWJ-MSCs-CM
FBG (mmol/L)	6.32 ± 0.71	32.2 ± 2.04^****^	21.80 ± 1.89^**** ##^
UAE (mg/24 h)	0.15 ± 0.01	65.74 ± 7.10^****^	17.48 ± 0.70^* ####^
ACR (mg/g)	0.41 ± 0.06	396.30 ± 45.06^****^	50.14 ± 3.52^**** ####^
Urine Output (ml/24 h)	7.53 ± 0.74	135.60 ± 13.13^****^	61.50 ± 13.27^** ###^
Water intake (mL/24 h)	23.13 ± 0.99	121.40 ± 4.14^****^	63.13 ± 7.39^**** ####^
Food intake (g/24 h)	14.50 ± 1.10	29.83 ± 2.02^****^	22.08 ± 1.20^** ##^
Body weight (g)	266.53 ± 3.30	167.90 ± 4.60^****^	241.03 ± 6.91^**** ####^

Data are expressed as mean ± SEM (n = 10)

^*^P < 0.05, ^**^P < 0.01 and ^****^P < 0.0001 represent significant difference with Control group

^##^P < 0.01, ^###^P < 0.001 and ^####^P < 0.001 represent significant difference with DM group

DM = Diabetic mellitus; hWJ-MSCs-CM = Conditioned media derived from human Wharton’s jelly mesenchymal stem cells;

FBG = Fasting blood glucose; UAE = Urine albumin excretion; ACR = urine albumin/creatinine ratio

### Effects of hWJ-MSCs-CM on liver enzymes, lactate dehydrogenase, and total bilirubin

**Fig. 4:**
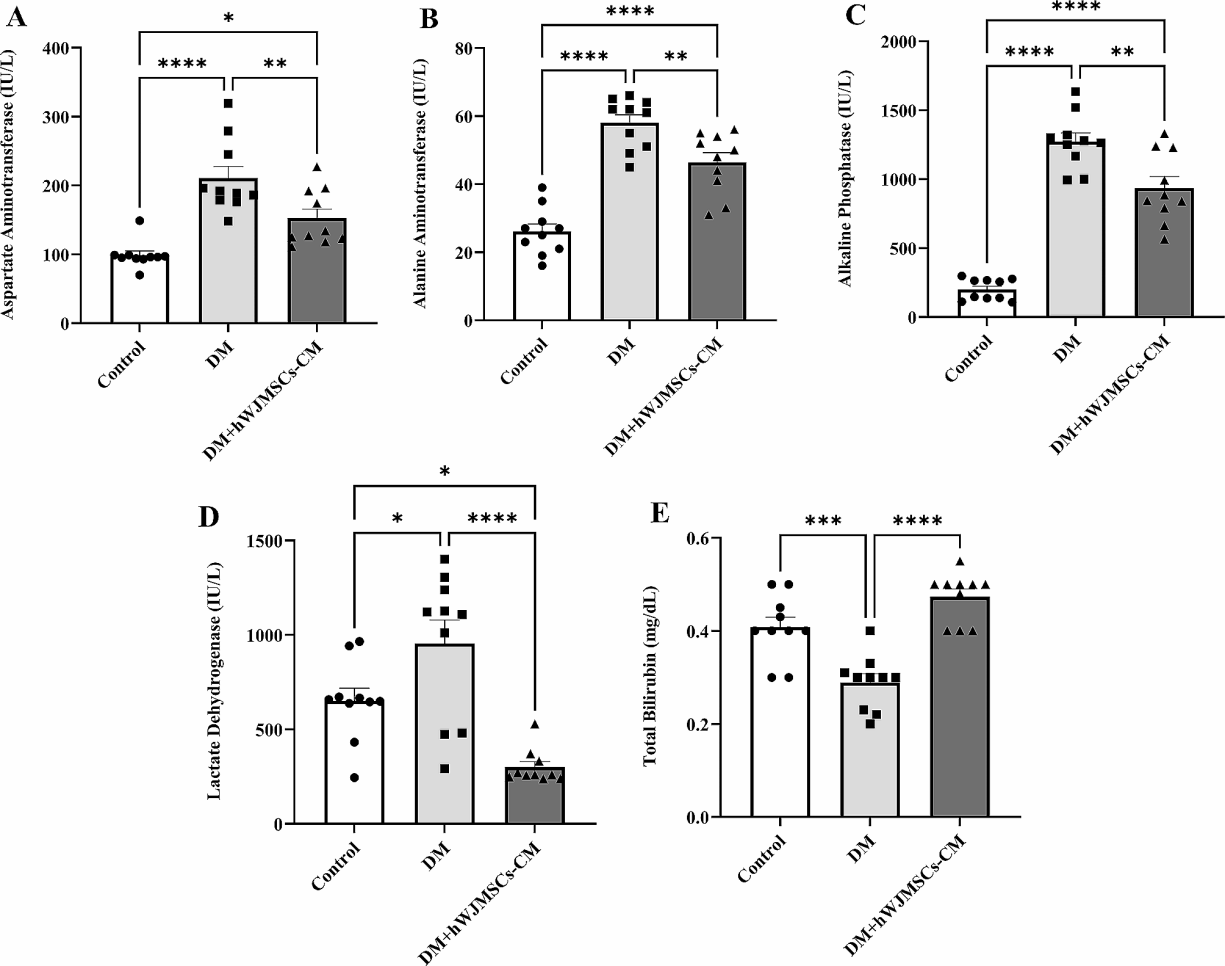
The effects of three weeks of hWJ-MSCs-CM treatment (500 µL per rat, i.p.) on serum activity of AST (**A**), ALT (**B**), ALP (**C**), LDH (**D**) enzymes, and total bilirubin (**E**) in rats with DM. Data are expressed as mean ± SEM (n = 10). ^*^P < 0.05, ^**^P < 0.01, ^***^P < 0.001, and ^****^P < 0.0001 represent significant differences between experimental groups. DM = Diabetic Mellitus; hWJ-MSCs-CM = Conditioned media derived from human Wharton’s jelly mesenchymal stem cells; AST = aspartate aminotransferase; ALT = alanine aminotransferase; ALP = alkaline phosphatase; LDH = lactate dehydrogenase

Figure 4 shows the effects of hWJ-MSCs-CM on the activity of liver enzymes (ALT, AST, and ALP), LDH, and total bilirubin in diabetic rats. The activities of ALT, AST, and ALP enzymes in the DM group were significantly (P < 0.0001) increased compared to controls. The LDH activity slightly increased in the DM group compared to the control group (P = 0.038). Moreover, total bilirubin significantly (P = 0.0005) decreased in the diabetic group in comparison with the control group. In diabetic rats that received hWJ-MSCs-CM for 21 days, the activity of liver enzymes, LDH, and total bilirubin modulated in the serum samples.

### Effects of hWJ-MSCs-CM on the Apelin and TGF-β genes expression in the kidney and liver tissues

**Fig. 5:**
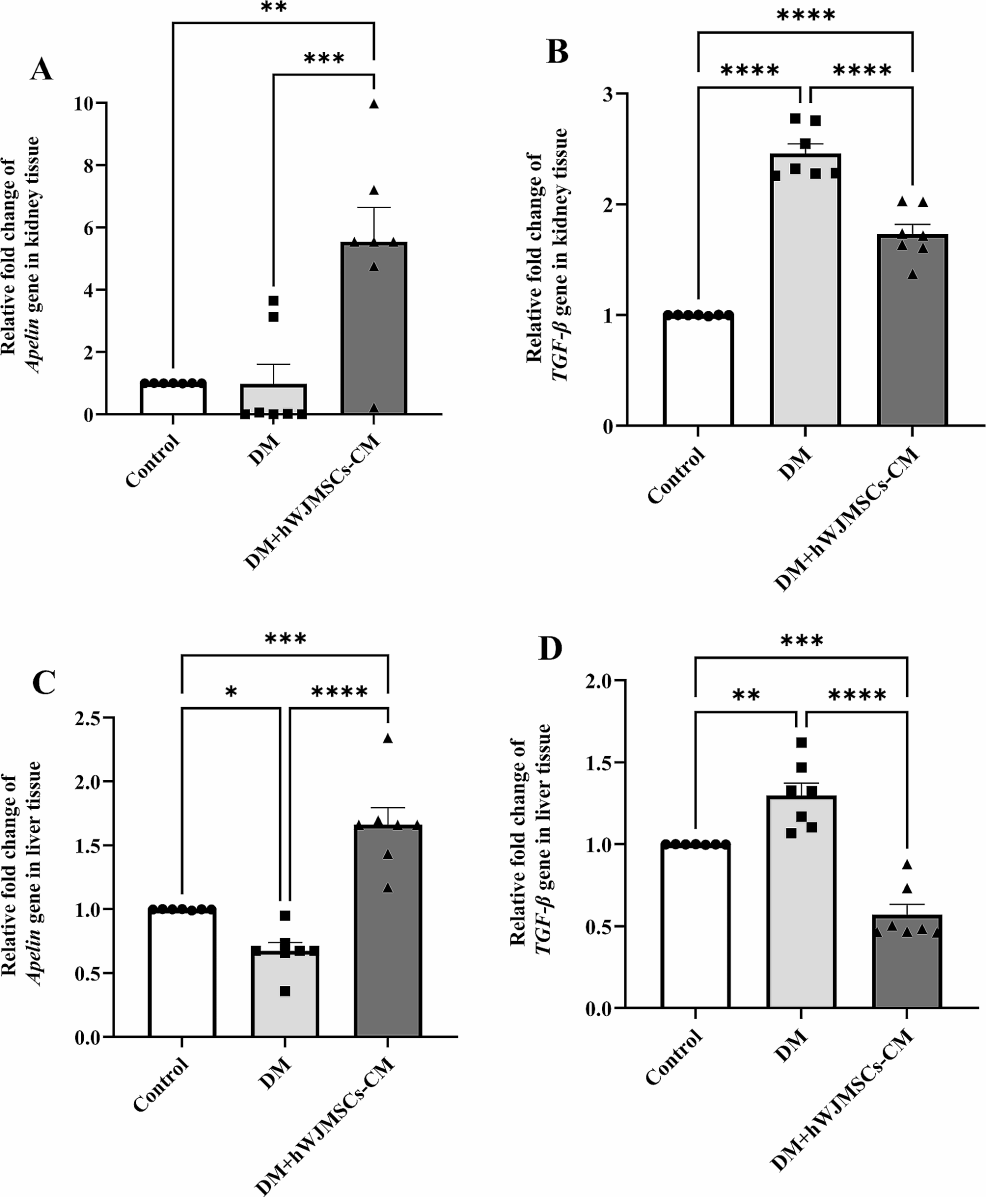
The effects of three weeks of hWJ-MSCs-CM treatment (500 µL per rat, i.p.) on gene expressions of apelin and TGF-β in the kidney (**A** and **B**) and liver (**C** and **D**) in rats with DM. Data are expressed as mean ± SEM (n = 7). ^*^P < 0.05, ^**^P < 0.01, ^***^P < 0.001, and ^****^P < 0.0001 represent significant differences between experimental groups. DM = Diabetic Mellitus; hWJ-MSCs-CM = Conditioned media derived from human Wharton’s jelly mesenchymal stem cells; TGF-β = transforming growth factor-beta

Apelin and TGF-β expression were examined in the kidney and liver tissues. Our data in kidney tissue showed that the mRNA level of apelin didn’t change in the diabetic rats compared to the basal expression in the control group. However, the TGF-β gene significantly (P < 0.0001) upregulated in the DM group compared to the controls. Treatment with hWJ-MSCs-CM significantly (P = 0.001) upregulated apelin expression compared to control and DM groups. TGF-β mRNA expression was downregulated in the DM + hWJ-MSCs-CM group compared to the DM group (P < 0.0001); however, this group still showed a significant difference (P < 0.0001) compared to controls (Fig. 5A and B).

On the other hand, our data in liver tissue showed that apelin gene expression was significantly (P = 0.040) downregulated, whereas TGF-β expression was significantly (P = 0.004) upregulated in the DM group compared to controls (P < 0.05). Treatment with hWJ-MSCs-CM significantly enhanced apelin expression (P < 0.0001) and diminished TGF-β expression (P < 0.0001) compared to the DM group. Interestingly, apelin gene expression was significantly increased, whereas TGF-β expression was significantly decreased in the DM + hWJ-MSCs-CM group compared to controls (P = 0.0001) (Fig. 5C and D).

### Histopathological analysis

#### Pancreas histopathological study

**Fig. 6:**
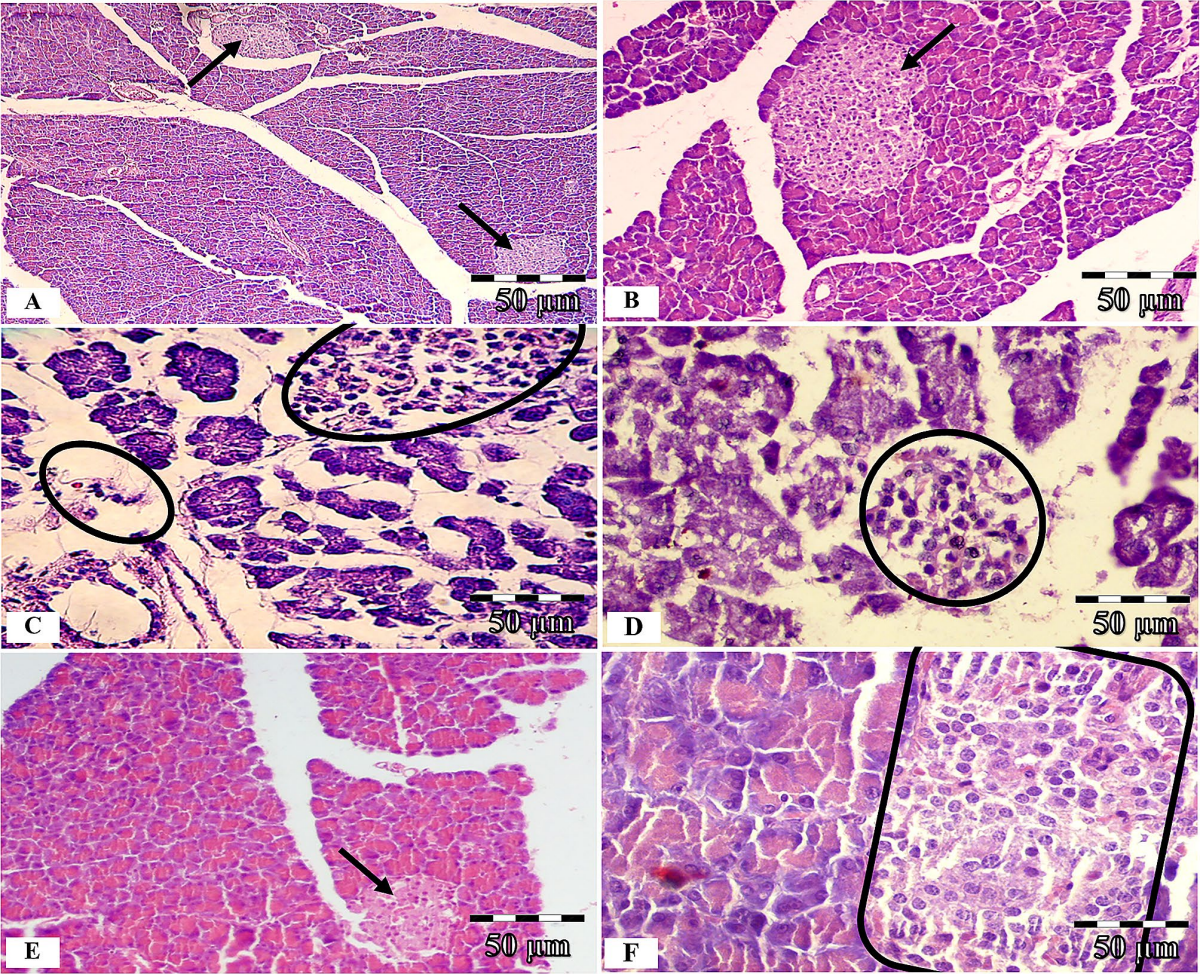
Hematoxylin and Eosin (H & E) staining of pancreas tissue; photomicrographs of the control, diabetic (without treatment), and diabetic rats treated with condition media (CM) of human umbilical cord Wharton’s jelly mesenchymal stem cells (hWJ-MSCs), [×200 and ×400 magnifications; Scale bar: 50 μm]. Microscopic views of the pancreas of control rats (**A** and **B**); diabetic rats with severe necrotic changes in the islets (black circle) and severe atrophy of the acinar parts (**C** and **D**); diabetic rats treated with condition media without any specific pathological changes except a mild decrease in the islet cells number and diameter (**E** and **F**)

In the control group, the islets of Langerhans were observed to be large and regular, and their limits were identified well. The cells of Langerhans islets and acinar parts were found to have a normal histologic appearance (Fig. 6A and B). In the DM group, a decrease in the number of pancreatic islets and their mean diameter, degenerative and necrotic changes, and subtle invasion of the immune cells in the parenchyma of the pancreatic islets were detected. Moreover, significant cell loss, nuclear changes, and karyolysis were observed in the islets; as the cellular order was disrupted, the islets were atrophied, the structure deteriorated, and in some places, residues of dead cells were visible. In addition, severe atrophy in the acinar parts of the pancreas was detected (Fig. 6C and D). A significant improvement was observed in the islet of Langerhans and acinar parts of the hWJ-MSCs-CM-treated group. Degenerative and necrotic changes were formed in only several cells in the DM + hWJ-MSCs-CM group (Fig. 6E and F).

At histomorphometric analysis, a significant decrease (P = 0.0006) in the number of pancreatic islets was observed in the DM group compared to the control group. Despite the significant difference (P = 0.002) with controls, the number of islets in diabetic rats treated with condition media showed a significant increase (P = 0.032) compared to the DM group (Table 3).

The pancreatic islets’ mean diameter was significantly decreased in the DM group compared to the control group (P < 0.0001). Diabetic rats treated with the condition media had a significantly higher mean islet diameter compared to the DM group (P = 0.003) and did not show any significant difference with controls (Table 3).

#### Kidney histopathological study

**Fig. 7:**
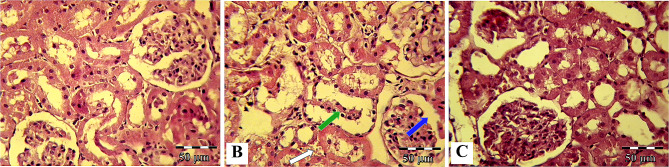
Hematoxylin and Eosin (H&E) staining of kidney tissue; photomicrographs of the control, diabetic (without treatment), and diabetic rats treated with condition media (CM) of human umbilical cord Wharton’s jelly mesenchymal stem cells (hWJ-MSCs), [×400 magnifications; Scale bar: 50 μm]. Microscopic views of the kidney of control rats (**A**); diabetic rats with marked dilatation of the urinary space (blue arrow), interstitial hemorrhage (white arrow), and mononuclear infiltration (green arrow) (**B**); diabetic rats treated with condition media without any specific pathological changes except partial hyperemia and slight swelling of tubular cells (**C**)

Normal kidney microscopic views of the control group are shown in Fig. 7A. In the renal tissue of diabetic rats, dilatation of the Bowman’s space with the proliferation of mononuclear cells and interstitial hemorrhage was detected (Fig. 7B). In addition, quantitative histopathological scores significantly (P < 0.0001) increased in the DM group compared to controls (Table 3). Human WJ-MSCs-CM therapy significantly prevented the occurrence of the above pathological lesions in diabetic rats, so that structural disturbance and histopathological scores improved in the DM + hWJ-MSCs-CM group compared to the DM group (P < 0.01) (Fig. 7C; Table 3).

**Table 3:** Quantitative histopathological analysis of pancreas, kidney, and liver tissues in the experimental groups

Experimental Groups Histopathological Variables	Control	DM	DM + hWJ-MSCs-CM
**Pancreas tissue**			
Number of pancreatic islets	15.51 ± 1.82	5.23 ± 0.51^***^	9.93 ± 1.23^** #^
Mean diameter of islets (µm)	642.32 ± 74.50	258.62 ± 52.60^****^	566.23 ± 50.35^##^
**Kidney tissue**			
Dilatation of the urinary space	0.00 ± 0.00	3.2 ± 0.03^****^	1.2 ± 0.04^**** ##^
Interstitial hemorrhage	0.00 ± 0.00	2.8 ± 0.07^****^	1.3 ± 0.01^**** ##^
Mononuclear infiltration	0.00 ± 0.00	3.1 ± 0.03^****^	1.8 ± 0.02^**** ##^
**Liver tissue**			
Hepatic necrosis	0.00 ± 0.00	1.62 ± 0.04^****^	0.48 ± 0.03^**** ####^
Hepatic degeneration	0.00 ± 0.00	2.55 ± 0.05^****^	0.66 ± 0.02^**** ####^
Mononuclear cell inflammation	0.00 ± 0.00	1.51 ± 0.04^****^	0.55 ± 0.02^**** ####^

Data are expressed as mean ± SEM

^**^P < 0.01, ^***^P < 0.001, and ^****^P < 0.0001 represent significant differences with the Control group

^#^P < 0.05, ^##^P < 0.01, and ^####^P < 0.0001 represent significant differences with the DM group. DM = Diabetic Mellitus; hWJ-MSCs-CM = Conditioned media derived from human Wharton’s jelly mesenchymal stem cells

#### Liver histopathological study

**Fig. 8:**
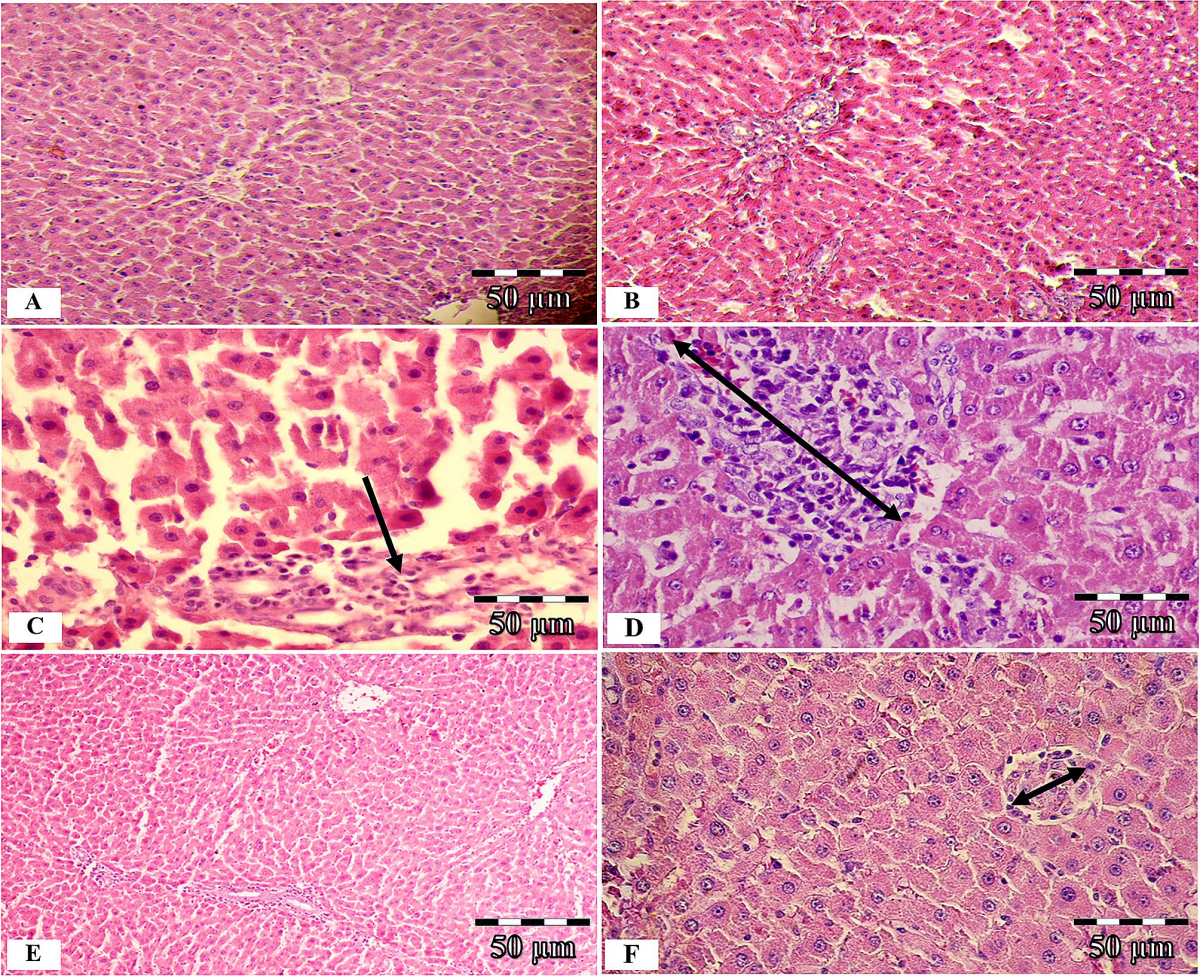
Hematoxylin and Eosin (H&E) staining of liver tissue; photomicrographs of the control, diabetic (without treatment), and diabetic rats treated with condition media (CM) of human umbilical cord Wharton’s jelly mesenchymal stem cells (hWJ-MSCs), [×200 and ×400 magnifications; Scale bar: 50 μm]. Microscopic views of the liver of control rats (**A** and **B**); section from the liver of the diabetic group with degenerated hepatocytes, karyolysed nuclei, extensive necrosis (**C**), mild fibrosis, bile duct proliferation (**C**, arrow), and lymphatic cell infiltration in parenchymal areas (**D**, two head-arrows); diabetic rats treated with condition media without any specific pathological changes except a mild degeneration and locally inflammatory cell infiltration (**E** and **F**)

Histological examinations of liver tissue are seen in Fig. 8. In the control group, liver tissue micrographs revealed normal hepatic cells, central vein regular architecture, and normal blood sinusoids (Fig. 8A and B). Extensive degeneration and necrosis in hepatocytes were seen in liver sections of the DM group. Large or small, irregularly-edged, partially rounded vacuoles were observed in the cytoplasm of degenerated hepatocytes. Mild fibrosis, bile duct proliferation (Fig. 8C, arrow), and inflammatory cell infiltration (Fig. 8D, arrows) were detected in parenchymal areas. The standard arrangement of hepatocyte cords was impaired and became an irregular cell community (Fig. 8C). These findings were found to be significantly decreased in the liver of rats in the DM + hWJ-MSCs-CM group (Fig. 8E). However, degeneration and inflammatory cell infiltration were observed locally in the hepatocytes (Fig. 8F).

In the diabetic rats treated with hWJ-MSCs-CM, histological scores, including hepatic necrosis, degeneration, and mononuclear cell inflammation, were substantially improved relative to the STZ-injected animals in the DM group (P < 0.0001) (Table 3).

## Discussion

Diabetes is a complicated syndrome with multiple organ disorders, such as liver and kidney, decreasing the quality of life and increasing mortality in DM patients [47]. The present study revealed the nephroprotective and hepatoprotective effects of human WJ-MSCs-CM for the first time in the STZ-induced diabetic rat model through apelin and TGF-β signaling pathways.

In the current study, a single dose of STZ was injected to create hyperglycemia, and this method has been widely used to induce animal models of diabetes, like the characteristics of human diabetes [48]. In this study, a histopathological assay of pancreas tissue in line with other works [42, 49] showed a decrease in the number of pancreatic islets and their mean diameter, degenerative and necrotic changes, and subtle invasion of the immune cells in the parenchyma of the pancreatic islets in diabetic rats.

Moreover, in agreement with other studies [50–52], our data demonstrated that the rats in the DM group progressed to severe hyperglycemia, microalbuminuria, polyuria, and polydipsia. Kidney injury was confirmed by functional and structural disturbances at the end of the 11th week. Diabetic nephropathy is a worldwide public health problem that involves one out of four diabetic patients and is a leading reason for end-stage kidney disease [53, 54]. Renal structural damages and increased plasma creatinine and GFR, which are characteristics of early diabetic nephropathy, indicated that a rodent model of diabetic nephropathy was successfully established.

In recent years, studies on treating diabetic complications by MSCs-CM have developed as a promising cell-free therapy due to the trophic effects of MSCs-secreted factors [55]. These trophic effects may improve renal structural changes, including glomerulosclerosis, tubular thickness, and intratubular proteinaceous casts in the cortex and outer medulla [56]. Our findings showed that hWJ-MSCs-CM injection improved kidney structural damage in the diabetic-treated group.

It has been confirmed that endothelial cell injury in the glomerulus tuft and enhanced glomerular permeability are the main factors in urinary albumin excretion [57]. Albuminuria is usually measured as an indicator of glomerular damage in diabetic nephropathy [58]. Based on our results, along with pathological changes, urine microalbumin, as well as urine albumin/creatinine ratio, was elevated in diabetic rats. At the same time, hWJ-MSCs-CM reduced albumin excretion in the diabetic group.

Diabetic liver injury is a common problem in diabetic patients [59]. In this study, liver functional changes were demonstrated by a significant increase in the serum levels of liver enzymes (AST, ALT, and ALP) and LDH, as well as a considerable increase in total bilirubin levels after DM induction. These changes in liver enzymes are confirmed by extensive degeneration and necrosis in hepatocytes. Other studies also showed that hepatic structural changes in STZ-induced diabetic rats were substantiated by increased AST and ALT activities [60, 61].

Reno- and hepato-protective mechanisms of the MSCs and MSCs-CM treatments from different sources have been investigated in several studies [31, 62]. Our result indicated that three weeks’ injection of hWJ-MSCs-CM improved liver and kidney structural changes and functional disturbances.

Apelin is a novel endogenous peptide recently recognized as a therapeutic target for glucose homeostasis and diabetic complications [32, 63], and TGF-β is a pleiotropic cytokine associated with progressive diabetic nephropathy and liver injury [11]. A correlation-based study indicates that the plasma levels of TGF-β1 are strictly related to the severity of renal disturbance in DM [12]. Based on human and animal studies, there is a close correlation between diabetes-induced renal and liver disorders and tissue expression levels of TGF-β [12, 64]. The current study findings also showed that mRNA levels of apelin and TGF-β were changed in the kidney and liver of diabetic rats. The apelin downregulation and the TGF-β upregulation have occurred in such a way. After three weeks of treatment of diabetic rats with hWJ-MSCs-CM, the expression levels of apelin and TGF-β were modulated, and the diabetic conditions were improved.

Recently, an interesting study transduced WJ-MSCs with apelin-expressing lentiviral particles, and those genetically modified cells were injected into the type 2 diabetes rodent model. Infusion of these WJ-MSCs-apelin significantly improved insulin sensitivity and increased the levels of plasma C-peptide. Moreover, the serum levels of inflammatory cytokines TNF-α and IL-6 decreased, and anti-inflammatory adiponectin levels increased [65].

## Conclusion

The present study showed that three weeks of treatment with hWJ-MSCs-CM could recover glycemic control via improved pancreas function. Free from cell therapy problems, CM therapy can ameliorate renal function by modulating microalbuminuria, creatinine levels, and GFR and liver function by regulating liver enzyme activities in diabetic rats. Finally, our results indicated that tissue regulation of apelin and TGF-β gene expression by hWJ-MSCs-CM also plays a crucial role in improving diabetic nephropathy and hepatopathy. Therefore, we suggest that hWJ-MSCs-CM can be further considered as a new protective approach against diabetic complications in the future.

## Data Availability

The datasets in the current study are available from the corresponding author upon reasonable request.
